# Instrumental Robots

**DOI:** 10.1007/s11948-020-00259-5

**Published:** 2020-08-19

**Authors:** Sebastian Köhler

**Affiliations:** Frankfurt School of Finance & Management, Frankfurt am Main, Germany

**Keywords:** Responsibility-gaps, Agency, Instruments, Human–robot collaboration, Responsibility

## Abstract

Advances in artificial intelligence research allow us to build fairly sophisticated agents: robots and computer programs capable of acting and deciding on their own (in some sense). These systems raise questions about who is responsible when something goes wrong—when such systems harm or kill humans. In a recent paper, Sven Nyholm has suggested that, because current AI will likely possess what we might call “supervised agency”, the theory of responsibility for individual agency is the wrong place to look for an answer to the question of responsibility. Instead, or so argues Nyholm, because supervised agency is a form of collaborative agency—of acting together—the right place to look is the theory of collaborative responsibility—responsibility in cases of acting together. This paper concedes that current AI will possess supervised agency, but argues that it is nevertheless wrong to think of the relevant human-AI interactions as a form of collaborative agency and, hence, that responsibility in cases of collaborative agency is not the right place to look for the responsibility-grounding relation in human-AI interactions. It also suggests that the right place to look for this responsibility-grounding relation in human-AI interactions is the use of certain sorts of agents as instruments.

## Introduction

Advances in artificial intelligence research—mostly advances in machine learning techniques combined with increased computing power—, allow us to build and employ fairly sophisticated automated systems: robots and computer programs like Google cars, military drones, or AlphaGoZero. It is natural to describe these systems—call them “current AI”—as *agents* who are capable of acting and deciding on their own, i.e. who are to some relevant degree autonomous (in some sense).^1^ One important question these systems raise is who is morally responsible when such AI perform actions with harmful outcomes (see e.g. Coeckelbergh 2016; Danaher 2016; Gunkel 2017; Hellström 2013; Hevelke and Nida-Rümelin 2015; Himmelreich 2019; Matthias 2004; Purves et al. 2015; Robillard 2018; Roff 2013; or Sparrow 2007).^2^ For those who think that current AI cannot themselves be responsible, this raises a challenge: one must identify a relation in which agents that *are* capable of being responsible stand to the outcomes of the AI’s action such that those agents are responsible for those outcomes. In a recent paper, Sven Nyholm (2018a) has argued that, because current AI will possess what one might call “supervised agency,” one should not consult the theory of responsibility for *individual* agency to find such a relation. Instead, because supervised agency is a form of *collaborative* agency—of *acting together*—one should consult the theory of collaborative responsibility—responsibility in cases of acting together.

This paper concedes that AI possess supervised agency, but argues that the relevant human-AI interactions are nevertheless not forms of collaborative agency and, hence, that the responsibility grounding relation in human-AI interactions is not to be found in the theory of collaborative responsibility. It also argues that if AI possess supervised agency, the place to find the relation that grounds responsibility in human-AI interactions is simply responsibility for the use of instruments. This is so, because if AI are supervised agents, this shows that the agency of the AI is not of any kind that would disallow us from subsuming responsibility in human-AI interactions under this explanation. Rather, there would be no in principle difference for questions about responsibility between AI and other supervised minimal agents, such as non-human animals, that are being used as instruments—for whom we know that responsibility explanations appealing to this relation are fitting.

The paper proceeds as follows: The first section (“Current AI, Agency, Responsibility Gaps”) states some of the assumptions that will be made in this paper and offers a more detailed presentation of the challenge current AI pose for questions about responsibility. The second section (“Nyholm’s Account: Human-AI interactions as Collaborative Agency”) presents in greater detail Nyholm’s argument for the thesis that to ground responsibility in human-AI interactions we should look at the theory of collaborative responsibility. The third section (“Human and AI: Not Working *Together*”) argues that supervised agency is *not* sufficient for collaborative agency and that, indeed, current AI can be expected *not* to possess the capacities required for collaborative agency, *even if* they possess supervised agency. Hence, Nyholm’s thesis that the theory of collaborative responsibility provides an account of the relation relevant for responsibility attributions in human-AI interactions, is false. The last section (“Instrumental Robots”) suggests an alternative of how responsibility can be grounded, if AI possess supervised agency. It argues that in this case, responsibility in human/AI interactions can just be subsumed under responsibility in *using something an instrument*.

## Current AI, Agency, Responsibility Gaps

This paper is concerned with ethical questions raised by certain automated systems, specifically certain robots and computer programs. What kinds of systems? This paper will be concerned with automated systems that can already or in the near future be built with machine learning techniques, where machine learning is the “field of study that gives computers the ability to learn without being explicitly programmed.”^3^ These systems can be expected to be capable of performing a certain range of fairly sophisticated tasks, such as driving, playing chess, scouting enemy territory, engaging enemy combatants, observing markets and making trading decisions based on these observations, and so on. They operate on various sorts of machine learning algorithms, which allow them to learn the operation of these tasks either in a supervised or an unsupervised fashion or through reinforcement learning (note not every program operating on a machine learning algorithm will, plausibly, be an agent; this paper will only be concerned with those that are). Importantly, systems that operate on machine learning algorithms do not perform their

core tasks—playing chess, assembling car parts, or selecting and engaging a target—according to a fixed procedure that was written out by human programmer. A robot that partly relies on learning algorithms has the ability to use data to modify the procedure by which it performs its core tasks. Depending on the degree of sophistication, learning algorithms enable a machine to sort through potentially very large, unstructured data sets and to extract from these data sets information that allows it to improve the rules it follows to perform its core tasks. (Burri 2017: 167/168)

Examples for such systems are e.g. autonomous vehicles, autonomous weapons systems, but also gaming programs like AlphaZero or certain sorts of financial trading programs.^4^

It seems very natural to describe such systems as *agents*, e.g. as “acting,” “deciding,” “intending,” “having the goal of,” “knowing,” “believing,” and so on. Indeed, doing so is very common in the philosophical literature concerned with moral issues raised by these systems (see e.g. Burri 2017; Coeckelbergh 2016; Danaher 2016; Gunkel 2017; Hellström 2013; Hevelke and Nida-Rümelin 2015; Himmelreich 2019; Purves et al. 2015; Matthias 2004; Roff 2013; or Sparrow 2007; see e.g. Robillard 2018 for a dissenting view). This paper simply grants that such systems are agents. However, it should be helpful to briefly say what will be meant by calling them “agents” and why one might think that they are agents.

Broadly speaking, agents are systems whose behavior can correctly be described as *intentional*, i.e. as appropriately connected to the system’s *intentions* (Davidson 1963). This means, in the first instance, that we can correctly ascribe at least some mental states that are relevantly related to the system’s behavior. However, this paper is not the place to explore the space of feasible options as to what this requires or argue for any one in particular (though it cannot be stressed enough that this is a very important and underexplored area of further research with regards to ethical questions about AI). Instead, the paper follows Nyholm in assuming a *functionalist* account of the mind and agency (another prominent view compatible with assigning mental states and agency to current AI would be an interpretative theory of mind (e.g. Dennett 1987, 1997)).

According to functionalist theories (e.g. Block 1980; Lewis 1972 or Putnam 1975; see Braddon-Mitchell and Jackson 1996 or Levin 2013 for excellent introductions into functionalist views about the mind), mental states are characterized by their causal-functional role in the system of which they are a part. Specifically,

a functionalist theory of mind specifies mental states in terms of three kinds of clauses: input clauses that say which conditions typically give rise to which mental states; output clauses that say which mental states typically give rise to which behavioural responses; and interaction clauses which say how mental states typically interact. (Braddon-Mitchell and Jackson 1996: 47)

For example, on such accounts, representational states are characterized by how they relate to sensory inputs, how they interact with other representational states, as well as how they combine with goal states to produce behavior. The representational state of a beer being in the fridge, for example, is characterized, among other things, by the fact that it is typically produced by a perception of the beer in the fridge and typically combines with a goal of having a beer to produce behavior such as going to and opening the fridge. Importantly, on functionalist views, if a system has states plausibly characterized by such causal-functional roles, this *suffices* for the system to have the relevant mental states. So, for example, if a system has a state that interacts with the system’s inputs, outputs, and other states as the given example of the representational state of a beer being in the fridge would, this is sufficient for the system to have this representational state. Importantly, it does not matter for functionalist views how these states are realized. In principle, a computer program could realize them just as well as a biological system.

Applied to agency, functionalist views suggest (e.g. List and Pettit 2011: 19–25) that a system’s behavior can be correctly described as intentional, if it has states that play the causal-functional role of *intentions*. A system plausibly has such states if it has (List and Pettit 2011: 20): (1) “representational states that depict how things are in the environment,” (2) “motivational states that specify how it requires things to be in the environment,” and (3) “the capacity to process its representational and motivational states, leading it to intervene suitably in the environment whenever that environment fails to match a motivating specification.” Given that current AI likely have causal-functional states satisfying these conditions, they are agents (especially given how easy it is to satisfy them. For a nice example that illustrates how easy it is to satisfy these conditions, see e.g. List and Pettit 2011: 19).

For example, even a system like AlphoGoZero likely has states with such causal-functional roles (at least after a suitable learning period). First, it will have states with the causal-functional role of states that depict how things are in the environment: what the state of the Go board is, for example, as well as what moves to take given the state of the Go board to achieve victory. These representational states are characterized by the fact e.g. that they change in certain ways to inputs to the system (when the system receives an input about the state of the Go board, it updates its representation of the state of the Go board), interact in certain ways with other representational states (for example, representations about the state of the Go board in combination of representation about how to achieve victory in Go interact to form representations about how to achieve victory given this state of the Go board), and so on. AlphaGoZero plausibly has exactly such states, given its capacities to process information about the state of any given Go game and how to win at it.

Second, AlphaGoZero will have states with the causal-functional role of states that depict how it requires things to be in the environment, most importantly, of course, that the state of the Go board be such that it has won the game. Such motivational states are, most importantly, characterized by the kinds of outputs they produce in combination with what representational states. Given that AlphaGoZero tends to make moves on the Go board that lead to victory given how it processes the state of the board, its plausible that it has at least the motivational state of aiming to win at Go.

Third, AlphaGoZero clearly has the capacity to process its representational and motivational states such as to intervene suitably in the environment whenever that environment fails to match a motivating specification. After all, AlphaGoZero will tend to make moves that will tend to lead to victory, given how it represents the state of the Go board. Hence, it seems that on a functionalist account, a system like AlphaGoZero would satisfy the three conditions relevant for agency.

Of course, within the set of entities with the capacity for agency so understood there are important further differences. One important difference is between agents who have, and agents who lack the capacities necessary for being responsible for their actions and the outcomes of those actions. Let us call the former “moral agents” and the latter “minimal agents.”^5^ Paradigmatic examples of moral agents are psychologically normal adult human beings. Paradigmatic examples of minimal agents are non-human animals and human children. Note that this already shows that there might be a lot of variety within these two classes of agents—different kinds of minimal agents, for example, might be capable of exercising agency of different degrees of sophistication.

This paper will take no stance on the question *what* capacities are required for *moral* agency and, hence, are lacking in minimal agents (as there is a substantial debate about this. See e.g. Darwall 2006; Fischer and Ravizza 1998; Frankfurt 2003; Kane 1996; Pettit 2001; Strawson 1962; Shoemaker 2015; Wallace 1994; or Wolf 1990). With regards to current AI, it will just be assumed, but not argued for (following the majority of philosophers writing on this issue) that current AI lack some of the relevant capacities required for moral agency and, hence, are not moral agents.^6^ There a good reasons to make this assumption (see e.g. Himma 2009; Purves et al. 2015; Sparrow 2007), but independently of this, questions of who is responsible when such AI perform actions with harmful outcomes are also only really interesting if they are not moral agents. After all, if these AI were moral agents, we should think about questions of responsibility for the AI’s actions in the very same way that we think about the responsibility for the actions of a psychologically normal adult human being.

With these remarks in place, the challenge current AI raise for questions about responsibility can be put more precisely. It is commonly assumed that current AI will be minimal agents that exercise a kind of minimal agency characterized by some degree of autonomy: it is assumed that these AI will be capable of acting (in some relevant sense) *on their own* to a significant degree. For example, we can expect there to be autonomous vehicles that will make independent decisions on what route to take, as well somewhat independent decisions on how to behave in certain traffic situations.^7^ However, some authors have argued that this creates a problem when such AI produce outcomes that are, on first sight, morally significant (see e.g. Danaher 2016; Gunkel 2017; Matthias 2004; Roff 2013; Sparrow 2007). This might occur, for example, when an autonomous weapons system produces an outcome that if it was produced by a human would be regarded as a war crime or when an autonomous car causes a seemingly avoidable accident. In such cases, the question arises who is responsible for the outcome. And the problem some authors see is that it seems that no one is responsible, although it seems that there ought to be someone who is responsible—there is a morally problematic so-called “responsibility gap.” First, the AI cannot be responsible—because (as was assumed here, following the majority of authors writing on this issue) it is not a moral agent and only moral agents can be morally responsible. But, second, because the AI exercises minimal agency characterized by some degree of autonomy, there is also no moral agent who can be responsible. This is so, because the agency of the AI interferes with any sort of relevant relation in which a moral agent could stand to the outcome. Specifically, it is argued that the agency of the AI interferes with the *control* that moral agents have over the outcome or with the ability of moral agents to *foresee* the outcome. But, because control and the ability to foresee are necessary for responsibility, no moral agent can be responsible for the outcome in question.

To avoid this conclusion, one needs an account of the relation in which *moral* agents stand to the AI’s actions and the outcomes of those actions, such that those agents *can* be responsible for those outcomes when they are morally significant. What would be ideal is to find a model for a responsibility-grounding relation between moral and minimal agents we are already familiar with and on the basis of which we can plausibly understand the relation between humans and current AI. This brings us to Nyholm account.

## Nyholm’s Account: Human-AI interactions as Collaborative Agency

Nyholm’s argument proceeds as follows: First, he highlights, correctly, that before we can draw conclusions about responsibility in human–AI interactions, we need a better understanding of what *sort* of minimal agency current AI will actually possess. Specifically, he correctly points out that there is not just the difference between minimal and moral agency, but that also minimal agency comes in different kinds and degrees. And, to figure out what conclusions to draw about responsibility in human-AI interactions, one needs to know what kind of minimal agency current AI will possess.

Nyholm argues (2018a: 1209/1210) that the kind of minimal agency current AI possess is plausibly a form of supervised agency.^8^ This is

pursuing a goal on the basis of representations and in a way that is regulated by certain rules or principles, while being supervised by some authority who can stop us or to whom control can be ceded, at least within certain limited domains. (Nyholm 2018a: 1208–1210)

He then claims that this shows that current AI’s agency is a form of *collaborative agency*, i.e. of “doing things together with somebody else,” rather than *individual agency*, i.e. of “doing things on one’s own” (Nyholm 2018a: 1210). Specifically, Nyholm’s claims that when the relevant kinds of AIs act, they act *together* with the supervising human(s) to achieve certain outcomes. And, hence, to determine who is responsible when such an AI produces a harmful outcome, one should look to theories of collaborative responsibility—i.e. theories about who is responsible for the outcomes of two or more agents *doing something together*, rather than theories of individual responsibility. And, Nyholm thinks that consulting *these* theories will, indeed, allow us to properly ground responsibility in human-AI interactions.

Nyholm supports this thesis by showing that it conforms to our intuitions about responsibility for relevant cases of human-AI interactions and offers a systematic explanation why our intuitions are correct. Take, for example, the following cases involving autonomous cars (Nyholm (2018a: 1213) argues similarly for cases involving automated weapons systems):

Driver: “A human is travelling in an automated vehicle, with the car in “autopilot” or “autonomous” mode. The human is supervising the driving, and would take over, or issue different driving-instructions, if this should be needed.” (Nyholm 2018a: 1212)

and

Company: “A human is travelling in an automated vehicle whose performance is monitored by the designers and makers of the car, who will update the car’s hardware and software on a regular basis so as to make the car’s performance fit with their preferences and judgements about how the car should perform in traffic.” (Nyholm 2018a: ibid)

Nyholm argues that, intuitively, the *humans* are the responsible party in Driver and Company—i.e. would be responsible if something goes wrong, even though the AI is doing most of the work. He argues that we can explain this if these cases fall under collaborative responsibility, because these cases are structurally similar to certain cases of collaborative agency and responsibility—cases in which two agents do something together in which the agent doing most of the work is incapable of being responsible and the other party *is* the one responsible, even though, she is doing very little in bringing about the outcome. His example is

Adult/Child: “An adult and a child are robbing a bank together, on the adult’s initiative, with the gun-wielding child doing most of the “work”. The adult is supervising the child’s activities, and would step in and start issuing orders to the child, if this should be needed.” (Nyholm 2018a: ibid)

In what follows it will be conceded that current AI possess supervised agency. In fact, this paper assumes that Nyholm made significant progress for the debate about responsibility in human-AI interactions simply by clarifying what sort of minimal agency current AI possess and by drawing attention to the fact that current AI’s agency is, plausibly, going to be some form of supervised agency. It will also be conceded that the intuitions about who bears responsibility in Driver, Company, and Adult/Child are correct. However, the next section argues that supervised agency is insufficient for collaborative agency. After that an alternative account will be offered to capture and explain our intuitions about cases like Driver, Company, and Adult/Child.

## Human and AI: Not Working *Together*

Nyholm claims (2018a: 1210) that supervised agency is a form of collaborative agency and that the latter is “doing things together with somebody else.” To assess whether the first claim is true, one needs to understand what it is to do something together with somebody else: What does it take for two or more agents to *do something together*?

The phenomenon itself should be familiar: consider a case where two people go for a walk together and contrast it with a case in which two people walk next to each other by coincidence. Only the first case, but not the second, is a case of two agents *doing something together*. In the debate in the philosophy of action concerned with this phenomenon it is called “shared” or “joint” agency. Given that Nyholm calls collaborative agency a case of “doing thing together with somebody else,” and given the sources he cites, this must be what he has in mind (in what follows phrases such as “doing something together,” “working together,” “joint actions,” “shared agency,” “acting together,” “collaborative agency” will be used interchangeably to mean this phenomenon).

While the phenomenon itself is familiar, it is difficult to determine what it takes for two or more agents to be involved in shared agency. It is common ground amongst all accounts of shared agency that to genuinely do something together, the agents involved need a *shared intention*: they need to intend that *they* ϕ. However, different accounts disagree on what it takes to have such a shared intention (see e.g. Bratman 2013; Gilbert 1996; Searle 1995; or Toumela 2007). Unfortunately for Nyholm, though, some of the prominent accounts on this matter already do not fit the explanatory purposes of his account.

Take, for example, an account like Margaret Gilbert’s (e.g. 1996, 2008). On her account, two or more agents have a shared intention, only if the agents undertake normative commitments that oblige them to certain sorts of performances and, hence, entitle the others to those performances. If such an account was correct, no agent that was not a moral agent could ever be engaged in shared agency, because only agents with the capacities characteristic of moral agency can be subject to obligations.^9^ Hence, current AI could not even *in principle* be part of joint actions.

If one had to rely on something like John Searle’s (1995) account, on the other hand, this would also be problematic for Nyholm. On his account, shared intentions are a *biological primitive*. Specifically, on Searle’s account for two individuals to have a shared intention to ϕ, they must each individually have a “we-intention.” A we-intention is an intention that takes the form “*we* intend to ϕ.” So, for example, when two people take a walk together, each of them must have the intention “We intend to go on a walk.” However, we-intentions themselves are a feature of biological systems that cannot be understood in simpler terms, such as, for example familiar *individual* intentions with a specific content^10^: they are a distinctive biological phenomenon that is irreducible to anything more basic. As should be clear, the fact that on Searle’s account shared intentions are a *biological* phenomenon implies that current AI could not even *in principle* be part of shared actions. However, even if this part of the theory was removed, that we-intentions are supposed to be a *primitive* phenomenon makes it so that there will be no informative criterion that would allow us to determine in a clear and non-question-begging manner whether current AI are capable of having such intentions. But, Nyholm’s account is credible, only if we have good reasons to think that current AI have such intentions, something that could not clearly be delivered by Searle’s account.

It seems, therefore, that some of the prominent accounts of joint intention already spell trouble for Nyholm from the get-go. In fact, if Nyholm’s account is to be on the right track, an account of shared agency like Michael Bratman’s will likely be his best shot.^11^ According to Bratman (2013: 103), it is true for two agents, call them “Anna” and “Ben,” that *they* intend to ϕ, iff

(a) Anna intends that they ϕ and (b) Ben intends that they ϕ.
Anna intends that they ϕ in accordance with and because of 1a, 1b, and meshing subplans of 1a and 1b; Ben intends that they ϕ in accordance with and because of 1a, 1b, and meshing subplans of 1a and 1b.
1. and 2. are common knowledge between Anna and Ben.

As a brief clarification: what is meant by “meshing subplans” in (2) is that in cases of shared intention, “there is a way [they] could [ϕ] that would not violate either [their] subplans [of 1a and 1b] but would, rather, involve the successful execution of those subplans” (Bratman 1993: 106).

On Bratman’s view, when (1)–(3) are satisfied, Anna and Ben have a shared intention that makes it the case that their execution of their individual intentions leads them to ϕ *together.* This account seems sufficiently undemanding in the sense that it would at least *in principle* allow minimal agents to perform joint actions, while still being informative enough to allow reasonable discussion of Nyholm’s suggestion that human-AI interactions should be regarded as cases of joint action. The central question is, though, whether—with these conditions in view—it is plausible that supervised agency is sufficient for the agents to do something together. This is not the case, which can be shown using the kinds of cases relevant for discussion, i.e. cases in which AI exemplify supervised agency.

Consider again

Driver: “A human is travelling in an automated vehicle, with the car in “autopilot” or “autonomous” mode. The human is supervising the driving, and would take over, or issue different driving-instructions, if this should be needed.”

and

Company: “A human is travelling in an automated vehicle whose performance is monitored by the designers and makers of the car, who will update the car’s hardware and software on a regular basis so as to make the car’s performance fit with their preferences and judgements about how the car should perform in traffic.”

While the AI in Driver and Company exemplifies supervised agency, these are not, or at least do not need to be, cases of genuinely doing something together in the sense explicated above. For example, notice that to understand Driver, there is no need to read it in a way that requires the passenger and the vehicle each intends that *they* drive home. For example, one could also read it so that upon the driver’s command, the vehicle intends to drive to a certain destination, while the passenger intends to *use* the vehicle to get home. Even on this reading, the vehicle would do exactly the job we would want it to do. So, on a natural understanding of Driver, condition (1) is not satisfied, which also means that (2) and (3) are not satisfied.

Furthermore, it seems quite plausible that for *most* human-AI interactions that can be expected in the near future, conditions (1)–(3) will not be satisfied.^12^ This is so, because for most of the things AIs will be designed and used for, it will not be *necessary* that they engage in shared agency with the humans that use them for their purposes: what is required is only that the AI performs a relevant service for the human. While this might require *supervised agency*, this does not require that the AI and the human do anything together in a genuine sense. For example, for a Google car to do its job, it must just drive passengers to their destination, but not engage in the activity of *driving to that destination together* with the passengers: neither the driver, nor the car need to intend that *they drive to the destination* for the car to do its job. Hence, Nyholm’s own cases serve as counterexamples to the thesis that supervised agency is sufficient for collaborative agency.

At this point, someone might object that the wrong sense of doing something together has been looked at here. What is relevant in human-AI interactions is not simply doing something together, but acting together in the setting of a *hierarchical group*, i.e. within a certain sort of command structure.^13^ However, Bratman’s account is not the proper account for that phenomenon, given that it presupposes that the agents involved are positioned, in some relevant sense, symmetrically. But, the fact that supervised agency is not sufficient for acting together in *that* sense does not establish that it might not be sufficient for acting together in the setting of a *hierarchical group*.

This objection, however, is unsuccessful. Bratman’s theory *can* be extended to the hierarchical case, as suggested by Scott Shapiro (Shapiro 2014: 264–270).^14^ He suggests that Anna and Ben ϕ together in a hierarchical setting—a setting involving *authority*, just in case

(4) ϕ is a shared intentional activity.
(5) Either Anna has ϕ-authority over Ben or Ben has ϕ-authority over Anna.
(6) If either Anna or Ben has ϕ-authority over the other, then
  - The authority intends that the subject adopt the content of the orders as subplans and revise the subject’s subplans so that they mesh with the orders.
  - The subject intends to adopt the content of the authority’s orders as subplans and to revise his subplans so that they mesh with the orders.
  - (a) and (b) are common knowledge.

In this case condition (4) *is* the above account of shared agency—it encompasses conditions (1)–(3). It is just that condition

(2) Anna intends that they ϕ in accordance with and because of 1a, 1b, and meshing subplans of 1a and 1b; Ben intends that they ϕ in accordance with and because of 1a, 1b, and meshing subplans of 1a and 1b.

is modified by conditions (5) and (6): the only difference between the case of acting together in a hierarchical setting and the egalitarian case is the way the meshing of subplans is achieved—in the egalitarian case there is mutual responsiveness in the revision of subplans, while in the hierarchical case the subplans of the authority determine those of the subjects. But, *that* difference to condition (2) should hardly make a difference as to whether supervised agency is sufficient for doing something together. After all, supervised agency is already insufficient to guarantee that condition (1) holds.

In conclusion, supervised agency is *not* sufficient for acting together. Furthermore, it is unlikely that most of the relevant AI will possess the capacities required for acting together in the sense required by (1). Therefore, responsibility in collaborative agency is not the right place to look for an account of how responsibility is grounded in human-AI interactions.

## Instrumental Robots

Of course, if Nyholm’s suggestion about the relation that grounds responsibility in cases like Driver and Company is wrong, one still needs some way to account for the intuitions about these kinds of cases. After all, Nyholm’s suggestion did have the benefit of explaining our verdicts about those cases by appealing to structurally similar cases of collaborative responsibility like Adult/Child. However, there is a rather straightforward alternative explanation.^15^

Cases like Driver and Company exemplify a particular relation: they are cases in which moral agents *use* something *as an instrument*. As should clear, however, the relation “using as an instrument for” *is* very relevant for responsibility allocations: there already is a significant set of norms, for example, for how much care needs to be employed when using instruments for certain purposes, what must be done when one is e.g. selling something as an instrument for certain purposes or what risks one may impose when using something as an instrument. And these kinds of considerations ground responsibility when something goes wrong in the use of the instrument in question. For example, someone who attempts to achieve a goal using an indeterministic machine that imposes a severe risk of harm on an innocent person is responsible if this harm occurs.

Of course, Driver and Company are cases in which the instrument that is being used is an *agent*. However, if it is true what has been assumed for the purposes of this paper, namely that the instruments that are being used are minimal agents that exercise supervised agency in these cases, there is no reason to assume that the agency of the AI poses an obstacle to grounding responsibility in human-AI interactions in the relation of *using as an instrument*. After all, we already *do* have a significant set of norms that ground responsibility for cases in which *minimal* agents capable of supervised agency are used as instruments.^16^ Many non-human *animals* are minimal agents capable of supervised agency and are used as instruments for many relevant purposes: horses are used for travelling to a certain destination, dogs are used for finding drugs and guarding people’s property, cattle are used for plowing fields, cats are used for controlling the pest population, etc.^17^ And when humans use such animals as instruments, responsibility is, in fact, grounded in the relation of *using as an instrument*. Hence, given that there would be no relevant difference between human-AI interactions and cases where an animal is being used as an instrument once we concede that both AI and animals are minimal agents capable of supervised agency, we can simply appeal to the relation of *using as an instrument* to ground responsibility in human-AI interactions.

While a fully fleshed out account of the relevant norms that ground responsibility in such cases cannot be offered here, consider examples from cases in which minimal agents are used as instruments to see what kinds of norms are relevant here and how such an account would proceed in the case of human-AI interactions.^18^ Take, for example, what sorts of norms apply to those who breed and train domesticated animals to sell them for certain purposes, such as police dogs, race horses, etc. Here, there are clear norms that are relevant for determining whether such persons are responsible when something goes wrong in the animal’s use. For example, the animal must have been sufficiently well trained for the intended job and trained to guarantee a reasonable degree of control by the user. If the animal is potentially dangerous, it must be trained in a way that reduces risks, users must be informed about the risks, it must be ensured that users are well trained in using animal, and so on. And, obviously, the seller is required to check to a reasonable degree that these conditions hold. So, there are norms of due care, norms for maintaining reasonable control, epistemic norms regarding how much information needs to be gathered, etc. that apply to those who offer minimal agents as instruments for certain sorts of purposes. If the person breeding and training the animal did not satisfy these norms, then if something goes wrong, they will be responsible.

Similar considerations apply to those who design AI for the use as instruments: the AI must be trained sufficiently well for the intended job, a reasonable amount of control over its behavior must be guaranteed, safety measures must be installed to prevent expectable harm, designers must have checked to a reasonable degree that all requirements are met, and so on. Of course, while the analogy with animals is fruitful, one must bear in mind that in the case of AI, designers have *much more* control over the constitution and behavior of the AI and, hence, are subject to even stricter norms.^19^

Similarly, norms apply not just to those *creating* or *training* minimal agents for the use as instruments, but to those *using* minimal agents as instruments. Consider, for example, attack dogs used by the police or the military as instruments for a variety of tasks, amongst them apprehending and subduing suspects or guarding and defending locations. Those who use attack dogs are required to ensure that such dogs do not harm innocent bystanders and, obviously, may not intentionally use the animal to cause unnecessary harm (i.e. by commanding the animal to attack innocent people). Furthermore, if they use the dog as an instrument for purposes for which they can reasonably foresee risk of harm occurring, they are responsible if something goes wrong, if the risk is too high or the harm too great. They, consequently, need to be aware of potential risks their use of the animal incurs and need to ensure that the animal has been sufficiently trained for the purpose they intend to use it for and need to treat the animal in a way that does not increase the risk of harm.

Again, similar considerations then apply to those who use AI as instruments for certain sorts of purposes: they must do their best to ensure that the AI does not harm innocent bystanders and, obviously, may not intentionally use the AI to cause unnecessary harm. Users should not make modifications to the AI that increase the risk of harm, and so on. Again, of course, we must bear in mind that in the case of AI, users have *much more* control over the behavior of the AI and are more capable of assessing the risks of using the AI than in the case of animal and, hence, should be subject to even stricter norms.

Note that these kinds of norms for the use of minimal agents as instruments, plausibly, just derive from *general* considerations about responsibility for the use of instruments, such as how much control users and designers have over the way instruments are used and the outcomes such use produces, how risky use of those instruments would be, as well as how bad relevant outcomes might be, if they occur. For example, dog trainers and breeders have significant control over the outcomes produced by the use of dogs for various purposes, but the outcomes also could, potentially, be quite bad, such as an innocent person being attacked by the dog. Similarly, those who use dogs for various purposes are, for example, well aware of the kinds of risks they, thereby, undertake under what sorts of circumstances. For such reasons the relevant norms that matter for determining who is responsible for when something goes wrong apply.

Of course, to determine the specific content of the norms with regards to AI, a fuller account of such AI will be required. For example, we need a clearer picture of the ways we can exercise control over such AI, what kinds of risks using such AI involves, and so on. These are important questions for further interdisciplinary research involving both philosophers and computer scientists. However, while these are important issues to settle, it is important to highlight that there is no good reason to assume that AI as minimal agents who exercise supervised agency are *distinctively* different from other kinds of *non*-*agential* things we might use as instruments when it comes to explaining responsibility. For example, it seems very plausible that there is no *moral* difference—one that could matter for attributing responsibility—between imposing risk by using a minimal agent exercising supervised agency or a non-agential tool. There seems to be no moral difference relevant for assigning responsibility, for example, in using either an indeterministic machine or a dog to achieve an outcome, if in either case there is a five percent chance of an innocent bystander being harmed.^20^ And, given that the agency of AI is—under the current assumption—not distinctively different from the agency of the dog in this case, one should also assume that something similar holds for AI. Insofar as there are differences between cases in which moral agents use minimal agents as instruments and cases in which moral agents use non-agential instruments, these are not *normatively relevant* differences.

At this point, though, a certain objection needs to be addressed. Some authors hold that current AI pose a problem, because their agency interferes with user’s and designer’s ability to *foresee* the effects of the AI’s actions. It might be held that this poses a problem for thinking that we can explain responsibility in human-AI interactions by appeal to the relation of *using as an instrument*. However, this objection can be dealt with within the framework on offer, by drawing on the kinds of considerations that are relevant when responsibility is grounded in instrument use.^21^ A first thing that requires clarification is in what sense users and designers might sometimes be unable to *foresee* what the AI will do. There seem two interpretations of this, neither of which poses a problem.

First, it might be held that it is *impossible* for users and designers to predict what the AI will do. Cases where an AI is used in a way that makes this true are certainly possible, if the AI operates on a machine learning algorithm. In fact, machine learning algorithms have already been used to solve problems or perform tasks, where the behavior of the algorithm was impossible to predict and actually outside of the scope of normal human capabilities. A paradigm example, here, are chess engines that play at a level no human being would be capable of—and hence make chess moves impossible to predict by humans.

These sorts of cases are not problematic for the argumentation made here. While users and designers might not be able to predict what the AI will do, it is users and designers who *choose to use an AI to solve a problem or perform a task while knowing that it is impossible to predict whether and how the problem will be solved or task will be performed*. There are many cases where making such a choice is completely unproblematic, because while the results might be unpredictable, there is simply no chance for a harmful outcome—here, again, chess engines are a good example. In these cases, questions about responsibility will simply not arise. But, there are cases easily imaginable where this is not the case, but where it is also pretty clear that the unpredictability of the outcome does not create an issue when it comes to determining who is responsible. Choosing an armed robot to perform a task such as engaging enemy combatants while *knowing that it is impossible to predict whether and how the robot will complete this task* is, quite simply, *reckless*. Someone who designs or uses military robots of this kind would, hence, *clearly* be responsible if a bad outcome occurs. So, for AI of this kind there is always the relevant question what kind of use it is put to and whether using the AI for such purposes bears a potential risk (and how large the risk is). But, all of these issues put the responsibility of designers and users square on the table, rather than interfering with their capacity for being responsible.

Of course, not all cases in which current AI are used are such that it is *impossible* for users and designers to predict what the AI will do. For example, those who design the AI that runs an autonomous vehicle will, obviously, aim to find and use algorithms that can be trained to *reliably* achieve certain expectable results and they will test and modify the AI until they can be reasonably confident that the product is *reliable*, i.e. predictably and robustly yields the results they are aiming for. Here, though, comes in the second sense in which users and designers might not be able to *foresee* what the AI will do. In this sense, users and designers might be incapable of fully ruling out the possibility of a harmful result, even for reliable AI. While this is true, it also does not pose a problem. After all, this sort of unpredictability is given *whenever* an instrument is used for a purpose that bears a risk for a harmful result. Even a reliable attack dog, for example, might act in harmful ways trainers and users cannot rule out. In such cases, users who know a risk is involved (and those who use potentially dangerous AI who satisfy their epistemic duties *will* know of such risks), often bear the responsibility if something goes wrong. This, of course, depends e.g. on how high the risk and how bad the outcome is that might occur, as well as on whether designers have satisfied the norms that apply to them. In cases, though, where it is genuinely the case that neither users nor designers of AI *are* plausibly responsible—because the risk involved was too low, the outcome not sufficiently bad, everyone satisfied duties of due care, and so on, it will be unfitting to think of them as giving rise to a responsibility gap. Just as with other instruments, such cases should be treated as *accidents*, where it is *proper* to think that no one is responsible. Hence, no matter how we understand the suggestion that there are cases in which users and designers are not be able to *foresee* the AI’s behavior, thinking of the relationship between users, designers, and AI as instances of the relationship between minimal agents that are used as instruments by moral agents allows us give adequate answers to questions about responsibility.^22^

So, the suggestion here is that to deal with cases like Driver and Company one can and should look at considerations regarding the responsibility for the use of instruments, specifically, the use of minimal agents capable of and engaging in supervised agency as instruments. Indeed, these considerations *seem* to yield the correct verdicts for Driver and Company: because the humans are the users or designers of the relevant instrument in these cases, it is those humans who are the responsible parties. What they are responsible for is, thereby, determined by the relevant degree of control, as well as duties of care, the risk involved in using the instrument, etc. that they have in virtue of their roles as users and designers of the relevant AI *as instruments.* In this respect, cases like Driver and Company should not be treated differently from other cases of instrument use, such as, for example, cases in which non-human animals are used and trained as instruments.

In fact, it seems that the explanatory power of this suggestion extends even to cases such as Adult/Child: it seems quite plausible that the best explanation of why responsibility is attributed as it is, should appeal to the fact that the child is used as an instrument by the adult. Of course, Adult/Child brings in additional complications compared to e.g. human-AI interactions. For example, the kind of control adults have over children is quite different from the kind of control users and programmers of AI have (the adult has e.g. *less* control over the actions of the child than the driver has over the actions of the automated vehicle, because the child has its own goals independently of those given to it by the adult). Additionally, children might raise other moral issues not present in the case of AI. For example, even if they are not moral agents, children’s interests are plausibly relevant from a moral point of view, while current AI likely do not have morally relevant interests. These kinds of issues make situations like Adult/Child more complex and messier from a moral perspective than human-AI interactions, as they raise additional moral questions. However, these differences do not make a difference to the aptness of the account on which the adult is the responsible party in Adult/Child, because the child is a minimal agent that is used as an instrument for the goals of the adult.

In conclusion, it seems that cases like Driver and Company can be accommodated by the view that the relation that grounds responsibility in human-AI interactions just *is* an instance of the relation “using as an instrument”—a view there is no reason to reject if one considers the agency of current AI to be supervised agency. Hence, once the view that the agency of current AI is supervised agency is on the table, we have a plausible explanation how responsibility is grounded in human-AI interactions. But, the explanation for how supervised agency grounds responsibility should appeal only to the familiar relation “using as an instrument”, and not to the idea collaborative responsibility.

## Footnotes

1 In what sense are these systems autonomous? As I will use the term, they are “autonomous” in the sense used in robotics, as being systems that operate and perform their given tasks more or less independently from human interference or intervention (e.g. US Department of Defense 2012 or Beer et al. 2014), but not “autonomous” in any more robust sense e.g. the sense that figures in Kantian moral theories (see e.g. Albertzart 2017). Thanks to a referee for *Science and Engineering Ethics* for requesting clarification.

2 This paper is concerned only with *moral* responsibility and brackets questions about other forms of responsibility (e.g. legal responsibility). Any reference to “responsibility” in what follows should be taken to be to moral responsibility. The paper also only focuses on responsibility for *harmful* outcomes, because it is not clear whether responsibility concerns regarding good and bad outcomes are symmetrical (for example, Wolf’s (1990) account suggests that these require different kinds of control). If such issues *are* symmetrical, everything argued here applies to responsibility for outcomes *in general*, but this paper stays uncommitted on that question. Thanks to the referees for *Science and Engineering Ethics* for requesting clarification on these points.

3 This popular definition is often attributed to Arthur Samuel’s seminal *Some Studies in Machine Learning Using the Game of Checkers* (1959), though he does not give that definition there verbatim. For introductory literature on the relevant concepts of machine learning, see e.g. Goodfellow et al. 2016 or Kuutti et al. 2019.

4 The debate concerned with responsibility and autonomous systems mostly focuses on two concrete kinds of AI applications (but see Matthias 2004 for an exception): autonomous weapons systems (e.g. Hellström 2013; Himmelreich 2019 or Sparrow 2007) or self-driving cars (e.g. Danaher 2016 or Hevelke and Nida-Rümelin 2015). Nyholm discusses both (Nyholm 2018a). Here the scope of what applications the discussion applies to is left intentionally vague, for the argumentation to be as general as possible. Those readers who are skeptical that a general approach is feasible for such a wide variety of possible applications should read the discussion as restricted only to the narrower set of applications Nyholm discusses.

5 The term “minimal agents” for the relevant kinds of agents comes from Himmelreich (2019).

6 Note that to assume that current AI are not moral agents is not to assume that there could not be AI that are. Note also that current AI lacking some of the relevant features for moral agency is compatible with them having or being capable of having some such properties, so that they might be moral agents in some weaker sense (even if they are not moral agents in the sense used here). For example, they might be what Moor (2006) has called “explicit ethical agents,” agents capable of ethical decision-making, without being what he calls “full ethical agents,” which is equivalent to what is called here “moral agents” (see e.g. Anderson and Anderson (2007) who argue that current AI could be explicit ethical agents).

7 An anonymous referee rightly points out that not all autonomous vehicles will have such a high degree of autonomy. Some might only have the capacity for lane correction or auto-stopping. Even for systems that exercise autonomous agency only in such a narrow range of tasks, however, the same questions of responsibility can arise, so these sorts of complications will be bracketed here. The reader who is doubtful about this should consider the discussion restricted to current AI that exercise a high degree of autonomy, which one can surely expect to exist in the near future.

8 Nyholm (2018a, b: 1208) uses the label „domain-specific supervised and deferential principled agency.”.

9 It is important to highlight that on Gilbert’s account the obligations and entitlements involved in shared agency are not *moral* obligations or entitlements, but obligations and entitlements of a distinctive normative kind (Gilbert 2009: 178). However, this does not change that only agents with the capacities required for moral agency could be subject to those obligations. This is so because on Gilbert’s account those obligations are very similar to moral obligations, e.g. in the way in which one is open to criticism when one violates those obligations.

10 We-intentions are, hence, not the same as the individual intentions of two individuals that *they* ϕ.

11 Note that this highlights that Nyholm’s account is committed to substantial assumptions in the philosophy of action and mind. The account offered later on is not hostage to fortune in this way.

12 It will not be denied here that it might be *possible* for human-AI interactions to satisfy (1)–(3). For this, the AI would have to possess a functional architecture that allows it to satisfy (1). Whether this is feasible or what that would look like is something on which the paper remains uncommitted. What is relevant for the point here is only that even if this is possible, it is first unlikely to be the case for most of the relevant AI applications and second something that seems absent even in the cases Nyholm discusses.

13 Indeed, in other work, Nyholm (2018b: 4/5) states that he has in mind *collaborative agency in a hierachical setting*.

14 Note that Shapiro does not, ultimately, endorse Bratman’s account. However, Shapiro’s alternative is not going to be of much help to Nyholm, as it imposes requirements that are not guaranteed to be satisfied in cases of supervised agency. Shapiro weakens conditions (1) and (2), but still requires the participants to *accept* a shared plan, and just as we can easily think of Driver and Company in a way that leaves condition (1) unsatisfied, we can think of them in a way that this condition is not satisfied.

15 This section has significantly profited from the comments of a referee for *Science and Engineering Ethics* who has pressed the question how the suggested account is to be understood. Thanks to the referee.

16 In fact, Nyholm’s reasoning to determine who is responsible itself plausibly fits the idea that what is at issue is not “collaboration,” but instead that humans *use* AI as *instruments*. Take the questions he takes to be relevant for settling who is responsible when something goes wrong (Nyholm 2018a, b: 1214/1215): “Under whose supervision and control is a vehicle that is currently operating in ‘autopilot’ or ‘autonomous’ mode operating?”, “Who is currently able to start, take over, or, at least, stop the car?”, “Whose preferences regarding driving-style is the car conforming to while in ‘autopilot’ or autonomous’ mode?”, “Who is better situated to observe and monitor the car’s actual behavior on the road?”, or “Who has an understanding of the functioning of the car, at least on a ‘macro-level’?”. These are *exactly* the sorts of questions one would ask if something went wrong in the use of an instrument to identify the responsible party.

17 A similar idea—that we should focus on the use of minimal agents as instruments to ground responsibility—has been suggested for the case of *legal* responsibility by Asaro (2012) and Calo (2015, 2016).

18 The author thanks a reviewer for *Science and Engineering Ethics* for requesting a further fleshed out version of this suggestion.

19 At this point the thoughtful reader might want to raise a concern about what is said here about the control designers have, given that in some cases it will be difficult for designers to predict what the AI will do—especially when one is dealing with certain sorts of machine learning applications such as those based on deep learning (this concern will apply similarly to what will be said about users in a second). This concern is well taken and will be discussed further down.

20 Thanks to an anonymous referee for *Science and Engineering Ethics* for pressing the question whether on the account suggested minimal agency makes a distinctive *normative* difference to explaining responsibility and for the helpful example used here. The position taken here is that minimal agency does not make such a difference. While space does not permit to argue for this, the example makes this position very plausible: assuming we hold the risk fixed, for example, there really seems to be no *moral* difference between risk imposition by using a dog or a non-agential machine that would matter for responsibility.

21 See Himmelreich (2019) for a similar response to this worry. A sketch of a similar line of argument can also be found in Köhler et al. (2018: 63). Note that, as Himmelreich argues, taking this route does not mean that one has to give up the assumption that responsibility requires control.

22 Thanks to a referee for *Science and Engineering Ethics* for asking for a more detailed response to this worry.
